# Supplementary material for: Low-Concentration T-2 Toxin Attenuates Pseudorabies Virus Replication in Porcine Kidney 15 Cells
Source: Toxins (Basel). 2022 Feb 6;14(2):121. doi: 10.3390/toxins14020121 (PMC8876018; doi:10.3390/toxins14020121)
Supplement: Supplementary file 1 [file toxins-14-00121-s001.zip › toxins-1550949-supplementary.pdf]

Supplementary Materials: Low-Concentration T-2 Toxin Attenuates Pseudorabies Virus Replication in Porcine Kidney 15 Cells

Kuankuan Xiong, Lei Tan, Siliang Yi, Yingxin Wu, Yi Hu, Aibing Wang and Lingchen Yang

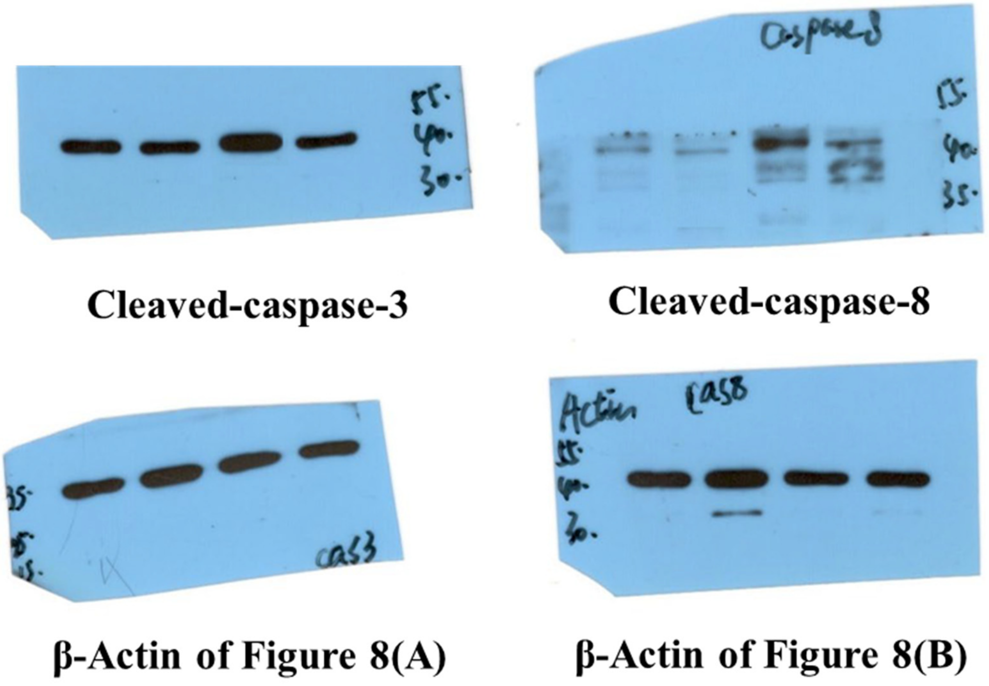

Figure S1. The full original scan of the Western blot results of Figure 8 in the article.

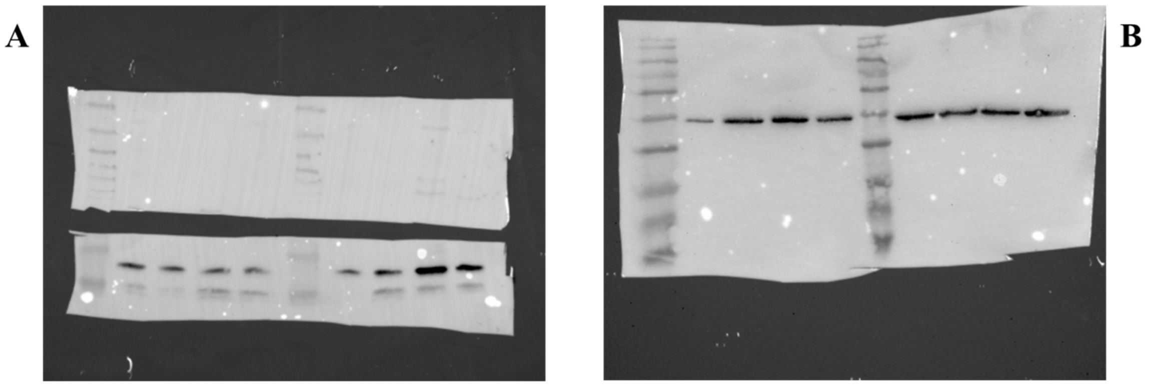

Figure S2. The full original scan of the Western blot results of Figure 8 in the article. (A) BAX and Bcl-2. (B) β-Actin in Figure 8-C.

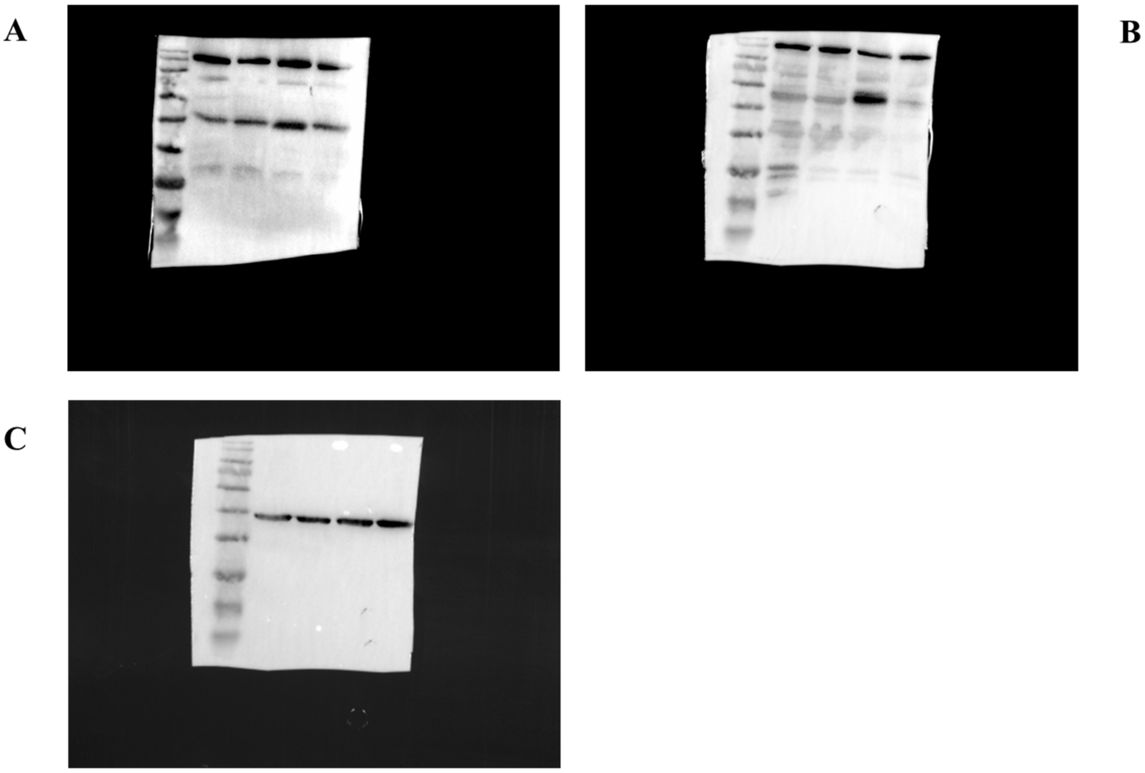

Figure S3. The full original scan of the Western blot results of Figure 8 in the article. (A) Gpx-1. (B) Nrf2. (C) β-Actin in Figure 8-D and E.
